# Foreign body reaction to bone wax an unusual cause of persistent serous discharge from iliac crest graft donor site and the possible means to avoid such complication - a case report

**DOI:** 10.1186/1757-1626-2-9097

**Published:** 2009-11-27

**Authors:** Abdul Qayum, Abid Hussain Koka

**Affiliations:** 1 Department of Orthopaedics, SKIMS Medical College, Srinagar, India

## Abstract

**Introduction:**

Bone wax is sometimes used in a variety of surgical procedures as a haemostatic. Bone wax contains beeswax softened with isopropyl palmitate or paraffin. It is nonabsorbable with no biochemical action. It achieves haemostasis by occluding the blood channels mechanically. Once applied it essentially never goes away. Bone wax reactions have been reported in literature many times.

**Case presentation:**

We report a case in which bone wax was used to control bleeding at the iliac crest from which bone graft was harvested. The foreign body reaction to bone wax caused persistent discharge from iliac crest graft donor site.

**Conclusion:**

Bone wax is a foreign body and that there is always a possibility of foreign body granulomas following its use. When necessary, bone wax should be used just for the time needed to achieve hemostasis. If it is left in place, care should be taken to avoid bone wax accumulation in the bony craters formed during surgery. Applying bone wax as a smooth layer may lead to this lumpy formation in the bony craters and one should be careful about it.

## Case presentation

A 43 year old female patient of Indian origin came to our hospital with nonunion fracture shaft of right humerus. She was treated elsewhere with closed reduction and U-slab for her fracture initially. Her fracture showed no signs of union even after 8 weeks of conservative treatment. Open reduction and internal fixation with narrow dynamic compression plate with cancellous bone grafting was done. Graft was taken from iliac crest right side.

She was discharged from the hospital 4^th ^day post surgery after inspecting her wound sites which were healthy. Suture removal was done on 11^th ^day.

**Figure 1:**
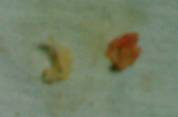
**Pieces of bone wax curetted out from the bony craters**.

**Figure 2:**
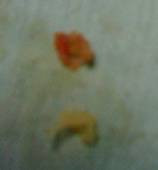
**Pieces of bone wax curetted out from the bony craters**.

She reported to hospital again on 14^th ^day with serous discharge from the graft wound. Her wound was alright with serous discharge coming out of a 3 mm hole in the wound. The edges were not indurated or red. Her ESR, CRP and blood counts were within normal limits. Culture of the discharge persistently was sterile. She was continued with dressings but the serous discharge was persistent and copious. Surgical exploration of the graft donor site was done. A thin layer of tissue was found at the graft donor site which was curetted out. Bone wax pieces were found in the craters at the iliac crest (Fig 1 &2) which was used at the time of bone grafting to control bleeding. The material was sent for histopathological examination which confirmed it as wax material. The wound of the patient healed normally with no discharge in future. Her fracture of humerus united well.

## Discussion

Bone wax was developed by Horsley in1886 [1]. It contains beeswax softened with isopropyl palmitate or paraffin. It is used in many surgical procedures to control bleeding. It is nonabsorbable with no biochemical action. It achieves hemostasis by occluding the blood channels mechanically [1]. Once applied it essentially never goes away. Surgeon should be aware of the adverse effects of its use. It is known to interfere with bone healing and osteogenesis. It has been shown to reduce bacterial clearance in cancellous bone and to increase the risk of infection. In the presence of bone wax, the number of bacteria needed to produce osteomyelitis is reduced by a factor of 10^4 ^(10,000) [2].

Foreign-body granulomatous reaction due to bone wax has been reported in various surgical sites with different clinical implications, requiring surgical exploration in some cases. Bone wax granulomas have been reported in mastoid [3], sternototomy site, lumbar disc surgical site [4], at the cerebellopontine angle [5], in the subarachnoid space near medulla oblongata, in femoral neck osteoplasty site [1], in orbits, in cranial defects, after tibial tubercle elevation surgery and after foot surgery. In one instance, in which bone wax had been used to stop bleeding from the iliac crest after the harvesting of autogenous graft, the patient presented 19 years later with a large, symptomatic, retroperitoneal tumour associated with a foreign-body reaction, which had to be removed operatively [6].

## Conclusion

Though inexpensive, easy to use with immediate effect on bleeding, bone wax should be used with caution after weighing the potential complications against the benefits. Bone wax is a foreign body and that there is always a possibility of foreign body granulomas following its use.

When necessary, bone wax should be used just for the time needed to achieve hemostasis. If it is left in place, care should be taken to avoid bone wax accumulation in the bony craters formed during surgery. Applying bone wax as a smooth layer by pasting with finger may lead to its lumpy formation in the bony craters and one should be careful about it.

## Consent

Written informed consent was obtained from the patient for publication of this case report and any accompanying images. A copy of the written consent is available for review by the Editor-in-Chief of this journal.

## Competing interests

The authors declare that they have no competing interests.

## Authors' contributions

AQ searched the literature and helped in editing. AHK conceived the idea, searched the literature and wrote the paper.
